# Effect of Age on NK Cell Compartment in Chronic Myeloid Leukemia Patients Treated With Tyrosine Kinase Inhibitors

**DOI:** 10.3389/fimmu.2018.02587

**Published:** 2018-11-08

**Authors:** Paulo Rodrigues-Santos, Nelson López-Sejas, Jani Sofia Almeida, Lenka Ruzičková, Patricia Couceiro, Vera Alves, Carmen Campos, Corona Alonso, Raquel Tarazona, Paulo Freitas-Tavares, Rafael Solana, Manuel Santos-Rosa

**Affiliations:** ^1^Faculty of Medicine, Institute of Immunology, University of Coimbra, Coimbra, Portugal; ^2^Laboratory of Immunology and Oncology, Center for Neuroscience and Cell Biology, University of Coimbra, Coimbra, Portugal; ^3^Faculty of Medicine, Center of Investigation in Environment, Genetics and Oncobiology - CIMAGO, University of Coimbra, Coimbra, Portugal; ^4^Department of Immunology, Instituto Maimónides de Investigación Biomédica de Córdoba - Reina Sofia University Hospital - University of Córdoba, Córdoba, Spain; ^5^Hematology Service, Coimbra Hospital and Universitary Centre, Coimbra, Portugal; ^6^Immunology Unit, University of Extremadura, Cáceres, Spain

**Keywords:** aging, CML, NK receptors, activation markers, differentiation markers, cytokines, NK cell subsets, tyrosine kinase inhibitors

## Abstract

Natural killer (NK) cells are a very important component of the innate immune response involved in the lysis of virus infected and tumor cells. Aging has a profound impact in the frequency, phenotype and function of NK cells. Chronic Myeloid Leukemia (CML) is caused by the BCR-ABL gene formation encoding aberrant oncoprotein tyrosine kinase. Treatment with tyrosine kinase inhibitors (TKIs) induces durable deep molecular response. The response to treatment and life expectancy is lower in older patients with chronic phase of CML than in younger patients. In this work we analyse NK cells from TKI-treated CML patients and healthy controls stratified according to age. We have analyzed the expression of NK receptors, activation markers, NK cell differentiation in CD56^bright^ and CD56^dim^ NK cell subsets and the expression of CD107a and IFN-γ in NK cells stimulated with K562. Whereas significant differences on the phenotype and function of NK cells were found between middle-aged (35–65 years old) and elderly (older than 65) healthy individuals, NK cells from TKI-treated CML patients do not show significant differences related with age in most parameters studied, indicating that age is not a limitation of the NK cell recovery after treatment with TKI. Our results also revealed differences in the expression of NK receptors, activation markers and functional assays in NK cells from TKI-treated CML patients compared with age-matched healthy controls. These results highlight the relevance of NK cells in TKI-treated patients and the need of an extensive analysis of the effect of aging on NK cell phenotype and function in these patients in order to define new NK-cell based strategies directed to control CML progression and achieve long-term disease remission after TKI cessation.

## Introduction

Natural Killer cells (NK) are innate lymphoid cells (ILCs) that represent ~15% of peripheral blood lymphocytes (PBLs). NK cells share many features with ILC1 although they are developmentally distinct (1). NK cells can be classified in two major subpopulations according to CD56 expression. CD56^bright^ NK cells are less differentiated subpopulation that represents < 10% of peripheral blood NK cells and have an immune-modulatory role with high production of cytokines and chemokines. CD56^dim^ NK cells, a more differentiated subpopulation that represents about 90% of NK cells, are mainly cytotoxic and interferon-gamma (IFN-γ) producers after direct contact with target cells (2, 3). A model of differentiation from immature CD56^bright^ that leads to more mature CD56^dim^ NK cells in the periphery has been proposed (4). Another subpopulation of NK cells, that do not express CD56 but express other NK receptors, expanded in healthy old and HIV-1 or hepatitis C infected individuals (5, 6), has been defined. NK cell function depends on a balance between activating and inhibitory signals triggered by activating and inhibitory receptors (7).

Chronic Myeloid Leukemia (CML) is an aging-associated disease (approximately half of cases are diagnosed in people older than 65) caused by reciprocal translocation between chromosomes 9 and 22 that give rise to the Philadelphia chromosome (Ph) and the BCR-ABL gene formation that encodes an oncoprotein tyrosine kinase with an aberrant activity in the hematopoietic stem cells (8, 9). Age has been included as a poor prognostic factor for survival in CML (10, 11). About half of the patients diagnosed with CML are between 60 and 65 years old (12–14) and the response to disease and life expectancy is lower in older patients with chronic phase of CML than in younger patients (15, 16). However, elderly CML patients are underrepresented in clinical studies having a reduced access to investigational therapies and median age of CML patients in cancer registries and patients included in clinical trials differs by 10–20 years (13, 14), supporting the interest to study immune parameters in elderly CML patients.

Immunosenescence is defined as age-associated dysregulation and dysfunction of the immune system characterized by impaired protective immunity and decreased efficacy of vaccines (17–20). These changes mainly affect the adaptive immune response (21) although consistent findings reveal that innate immune response is also affected (22, 23). In addition to age, situations of chronic activation of the human immune system, such as viral infections, autoimmune diseases and cancer, are involved in the development of immunosenescence (24–27).

Age-related alterations in frequency, distribution, phenotype, and function of NK cell subsets have been described, including an increased expression of CD57 (considered a marker of ‘memory-like' NK) and a decreased expression of Natural Cytotoxicity Receptors (NCRs) and other NK activating receptors (22, 23, 28–31), CD69 (32), and CD94/NKG2A, and an increase of killer Ig-like receptors (KIR) (33–35) in older individuals.

Several studies have also found a decrease in the frequency and function of NK cells in CML patients at the time of diagnosis, with a progressive functional deterioration during all phases of the disease (36–39). It has been described a decreased expression of NKG2A, NKp30, and NKp46 at the time of diagnosis and changes in the NKG2C and KIR receptors (40, 41). Patients with CML also show a decrease in NKG2D expression, that mediates NK anti-CML response through its ligands MICA/B, when compared with healthy controls (42). NK cells from acute myeloid leukemia patients also have a downregulated expression of activating receptors NKp30 and NKp46 (27, 43–45) and DNAM-1 (27, 46), likely as a consequence of the interaction with their ligands in leukemic blasts.

The standard treatment for CML patients is based in the use of tyrosine kinase inhibitors (TKIs) such as imatinib, and more potent second-generation nilotinib and dasatinib, that have improved CML poor prognosis (47, 48). TKIs have a direct effect inhibiting the BCR-ABL1 kinase activity to induce a durable deep molecular response, a prelude to successful treatment-free remission that occurs in ~50% of all CML patients who cease TKI therapy (48–51). In addition to their direct anti-kinase activity, TKIs contribute to the restoration of immune cell function leading to the efficient immunological control of CML (41). Recent studies show the impact of TKIs on NK cells, finding that the expression of NK activating receptors is restored to normal levels compared to their low level at the time of diagnosis (40, 41).

Considering that both aging and CML induce changes in phenotype and function of NK cells, in this work we have studied the expression of several markers (CD11b, CD27, CD57, CD69, HLA-DR, NKG2A, NKG2C, NKG2D, NKp30, NKp44, NKp46, and NKp80) in NK cell subsets, and CD107a and IFN-γ in K562 stimulated NK cells, from CML patients and healthy controls, stratified according to age in middle-aged and elderly donors.

## Materials and methods

### Study subjects

A total of 80 individuals were included in the study, 38 CML patients treated with first-line TKIs Imatinib, and 42 healthy controls, stratified in two groups according to age: middle-aged (35–65 years) and old-age (over 65 years) (Table 1). All the participants in the study were CMV-seropositive (Non-reactive IgM and reactive IgG, data not shown). Controls were excluded of the study if they had infection at the time of sample collection, suffered or had suffered cancer or autoimmune diseases, were under immunosuppressive drugs or calcium channel blockers. The Ethical Committees of the Faculty of Medicine of the University of Coimbra and the Coimbra Hospital and University Centre (Portugal) and the Ethics Committee of the Reina Sofia University Hospital of Cordoba (Spain) approved this study and all volunteers agreed and signed informed consent to participate.

**Table 1:** Demographics characteristics of individuals (*n* = 80).

**Group name**	**No**.	**Sex (male: female)**	**Mean (SD)^a^**	**p^b^**
Middle age (Control)	19	8:11	51 (7.85)	0.924
Middle age (CML^*^)	19	12:7	51 (8.97)	
OLD (Control)	23	10:13	74 (6.17)	0.814
OLD (CML^*^)	19	12:7	75 (5.71)	

a *Average age (Standard Deviation) of the group*.

b *p-value for the comparison of means between controls and CML, within the same age group (t-Student test)*.

* *TKI-treated CML patients*.

### Procedures of sample collection and processing

Peripheral blood samples from all individuals were obtained in heparinized tubes. Flow cytometry studies were performed on freshly-obtained cells. After antibody staining, BD FACS Lysing Solution (BD Biosciences, San Jose, CA, USA) was used for lysis of red blood cells. Subsequently, cells were washed and resuspended in Dulbecco's Phosphate Buffered Saline (PBS) pH 7.4 (Ambion, Austin, TX, USA) for later acquisition on the cytometer. Cell suspensions were acquired in a BD FACS Canto II cytometer (BD Biosciences, San Jose, CA, USA). These procedures were performed according to the manufacturer protocols.

### Flow cytometric analysis and monoclonal antibodies

Fresh blood was used for the analysis of the surface receptors by flow cytometry in different tubes. The following mouse anti-human conjugated monoclonal antibodies (mAbs) were used: anti-CD3 V500 (clone UCHT1, BD Horizon), anti-CD14 V500 (clone M5E2, BD Horizon), anti-CD19 V500 (clone HIB19, BD Horizon), anti-CD3 APC-H7 (clone SK7, BD Biosciences), anti-CD56 PerCP-Cy5.5 (clone HCD56, BD Pharmingen), anti-CD11b V450 (clone ICRF44, BD Horizon), anti-CD27 FITC (clone 0323, Biolegend), anti-CD57 Pacific Blue (clone HNK-1, Biolegend), anti-CD69 FITC (clone FN50, Biolegend), anti-HLA-DR V500 (clone G46-6, BD Pharmingen), anti-NKp30 Alexa Fluor 647 (clone P30-15, Biolegend), anti-NKp44 Alexa Fluor 647 (clone P44-8, Biolegend), anti-NKp46 PE (clone 9E2, Biolegend), anti-NKp80 PE (clone SD12, Biolegend), anti-NKG2A PE (clone 131411, R&D Systems), anti-NKG2C APC (clone 134591 R&D Systems), anti-NKG2D APC (clone 1D11, Biolegend). The expression of HLA-DR, NKp30, NKp44, NKp46, NKp80, NKG2D, (measured as Median Fluorescence Intensity, MFI) and the expression of CD11b, CD27, CD57, CD69, NKG2A, and NKG2C (measured as relative frequency) were determined in the different NK cell subpopulations and analyzed by multiparametric flow cytometry. The data were analyzed using FlowJo v10 (Tree Star, Inc.) from PBLs, selecting singlets. NK cells (CD56^+^ and CD3^−^/CD14^−^/CD19^−^) were selected and two subpopulations of NK cells were described, in relation to their expression of CD56, as CD56^bright^ and CD56^dim^ (Figure S1A). Isotype matched antibodies, labeled with the appropriate fluorochromes, were used as negative controls. Representative histograms for the NK cell markers, and the isotype and fluorochrome of the antibodies used in the studies, are shown in Figure S1B.

### NK cell stimulation with K562, degranulation assay, and IFN-γ staining

Effector peripheral blood mononuclear cells were isolated from heparinized blood samples by a standard density gradient procedure (Ficoll-Paque PLUS, Merck KGaA, Darmstadt, Germany) and counted to adjust the concentration to 50 × 10^6^cells/mL. Target cells (K562 cell line) were prepared; viability determined and counted to adjust the concentration to 1 × 10^5^cells/mL. Then, effector and target cells were mixed in a 25:1 effector-to-target ratio into 12 × 75 mm tubes with anti-CD107a PE (clone H4A3, BD Pharmingen®) antibody and brefeldin A (Merck, 10 μg/mL). Tubes were incubated in a humidified CO_2_ incubator in the water reservoir at the bottom for 4 h. At the end of incubation cells were washed and resuspended in 100 μL of 1xPBS (phosphate-buffered saline) and the extracellular antibodies were added, anti-CD56 APC (clone B159, BD Pharmingen®) and anti-CD3V500 (clone UCTH1, BD Horizon®). After 15 min of incubation at RT in the dark, suspensions were treated with Fix and Perm A solution (Invitrogen®) for 15 min, in the dark at room temperature. Cells were centrifuged at 453 g for 5 min and the supernatant discarded. Next, cells were incubated with Fix and Perm B solution (Invitrogen®) and the intracellular antibody anti-IFN-γ PE-Cy7 (clone B27, BD Horizon®) for 20 min, in the dark at room temperature. After centrifugation at 453 g for 5 min and supernatant discarded cells were resuspended in 1x PBS and acquired in the flow cytometer (BD FACS Canto II).

### Statistical analysis

Shapiro-Wilk test was used for checking the normal distribution of the data. We used Kruskall-Wallis (non-parametric) test for multiple comparison and Mann-Whitney *U*-test (non-parametric) was used to compare specific groups. All tests were performed using statistical software package SPSS 18.0 (SPSS Inc., Chicago, IL, USA). Values *p* < 0.05 were considered significant. The results were shown as median with interquartile range and the graphics were performed using GraphPad Prism software version 6.0 (GraphPad Software, La Jolla, CA, USA).

## Results

### Expression of activating and inhibitory receptors on NK cells from TKI-treated CML patients

**Figure 1:**
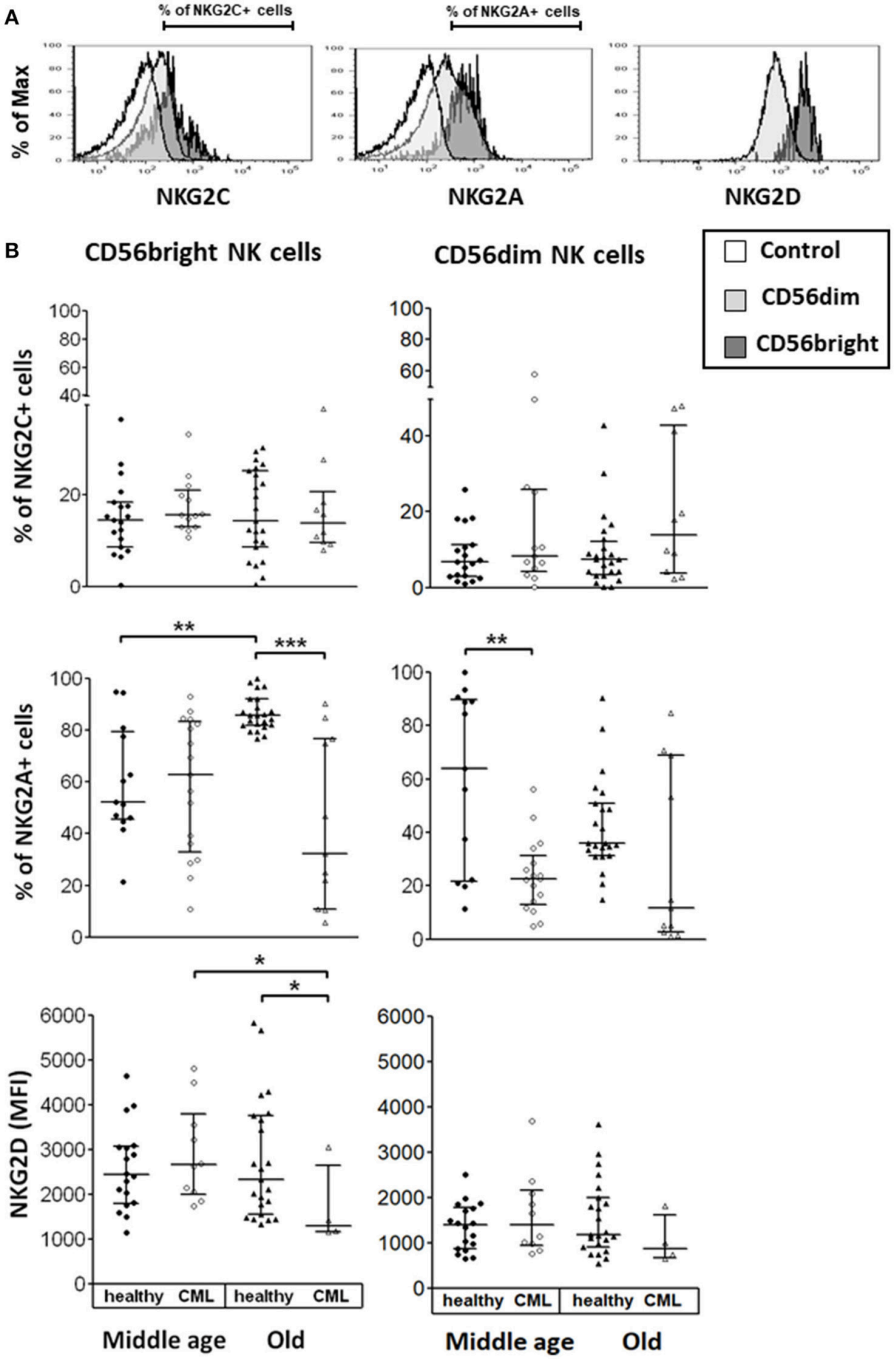
Expression of Natural Killer Group 2 (NKG2) receptors on NK cell subpopulations. **(A)** Representative histograms for each marker are shown (the non-shaded area represents the control, the shaded area of light gray, the CD56^dim^ cells, the shaded one of gray, the CD56^bright^ cells). The percentage of cells expressing NKG2C, NKG2A, and NKG2D as (MFI) measured in the total of cells, was determined on the surface of each subset by multiparametric flow cytometry. **(B)** Expression of activating receptors (NKG2C and NKG2D) and of inhibitory receptor (NKG2A) on CD56^bright^ and CD56^dim^ NK subsets from healthy individuals and TKI-treated CML patients, stratified according to age (middle-aged 35–65 years and old >65 years). Number of donors: NKG2C middle-aged healthy *n* = 19, middle-aged CML *n* = 13, old healthy *n* = 23, and old CML *n* = 10; NKG2D middle-aged healthy *n* = 18, middle-aged CML *n* = 10, old healthy *n* = 23, and old CML *n* = 4; NKG2A middle-aged healthy *n* = 13, middle-aged CML *n* = 17, old healthy *n* = 23, and old CML *n* = 11. The results, expressed as median with interquartile range, were considered significant at *p* < 0.05. *P-*values were determined comparing middle age with old and healthy with TKI-treated CML patients. ^*^p <0.05; ^**^p <0.01; ^***^p <0.001.

We studied the expression of activating and inhibitory receptors on CD56^dim^ and CD56^bright^ NK cells in healthy donors and CML patients, stratified in middle age and old age. The expression of Natural Killer Group 2 (NKG2) receptors was measured as the percentage of positive cells or as MFI measured in the total of cells (Figure 1A). Our results showed a significant decrease in the expression of the inhibitory receptor NKG2A on CD56^dim^ NK cells in middle-aged CML patients compared with middle-aged healthy donors and a decrease in the percentage of NKG2A^+^CD56^bright^ NK cells in old CML patients compared with old healthy donors. Age-associated changes in the expression of NKG2A were only observed in healthy donors showing an increase with age in the percentage of NKG2A^+^CD56^bright^ NK cells. In contrast, NKG2C receptor expression was not influenced by CML or age. Regarding the activating receptor NKG2D, we found a significant decrease in the MFI of NKG2D on CD56^bright^ NK cells in old CML patients compared with old healthy donors, and a decrease with age in CML patients (Figure 1B).

**Figure 2:**
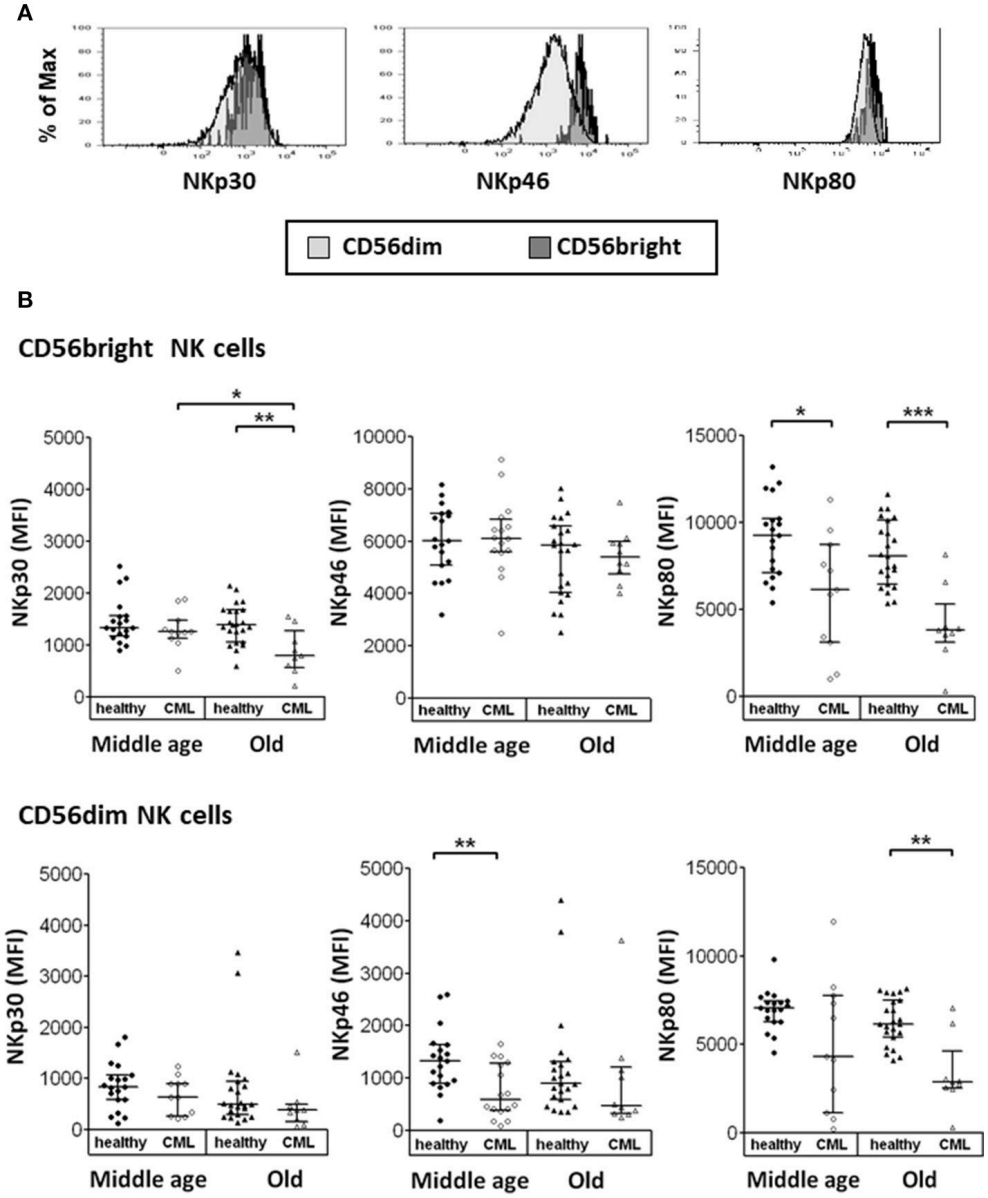
Expression of Natural Cytotoxicity Receptors (NCRs) on CD56^bright^ and CD56^dim^ NK cells. **(A)** Representative histograms for NKp30, NKp46, and NKp80 are shown (the shaded area of light gray, the CD56^dim^ cells, the shaded one of gray, the CD56^bright^ cells). Results were expressed as Median Fluorescence Intensity (MFI), measured in the total of cells. **(B)** Expression of activating receptors NKp30, NKp46, and NKp80 on CD56^bright^ and CD56^dim^ NK subsets from healthy individuals and TKI-treated CML patients, stratified according to age (middle-aged 35–65 years and old >65 years). Number of donors: NKp30 and NKp80 middle-aged healthy *n* = 19, middle-aged CML *n* = 11, old healthy *n* = 23, and old CML *n* = 9; NKp46 middle-aged healthy *n* = 19, middle-aged CML *n* = 16, old healthy *n* = 23 and old CML *n* = 10. The results were expressed as median with interquartile range. *P-*values were determined comparing middle age with old and healthy with TKI-treated CML patients and were considered significant at *p* <0.05. ^*^ *p* < 0.05; ^**^p < 0.01; ^***^p < 0.001.

We have also studied the expression of NCRs on NK cell subpopulations. Results were expressed as MFI measured in the total number of cells (Figure 2A). We have found that the expression of NKp30 and NKp80 (not a NCR) on CD56^bright^ NK cells and NKp80 on CD56^dim^ NK cells was lower in old CML patients compared with old healthy donors. NKp80 expression on CD56^bright^ cells and NKp46 expression on CD56^dim^ NK cells were significantly decreased in middle-aged CML patients compared with middle-aged healthy donors. The analysis of age-associated changes in the expression of NCRs in CML patients showed a decrease in NKp30 expression on CD56^bright^ NK cells. No significant differences associated with age (middle-aged vs. old age) were observed in healthy donors (Figure 2B).

### Expression of activation markers of NK cells from TKI-treated CML patients

**Figure 3:**
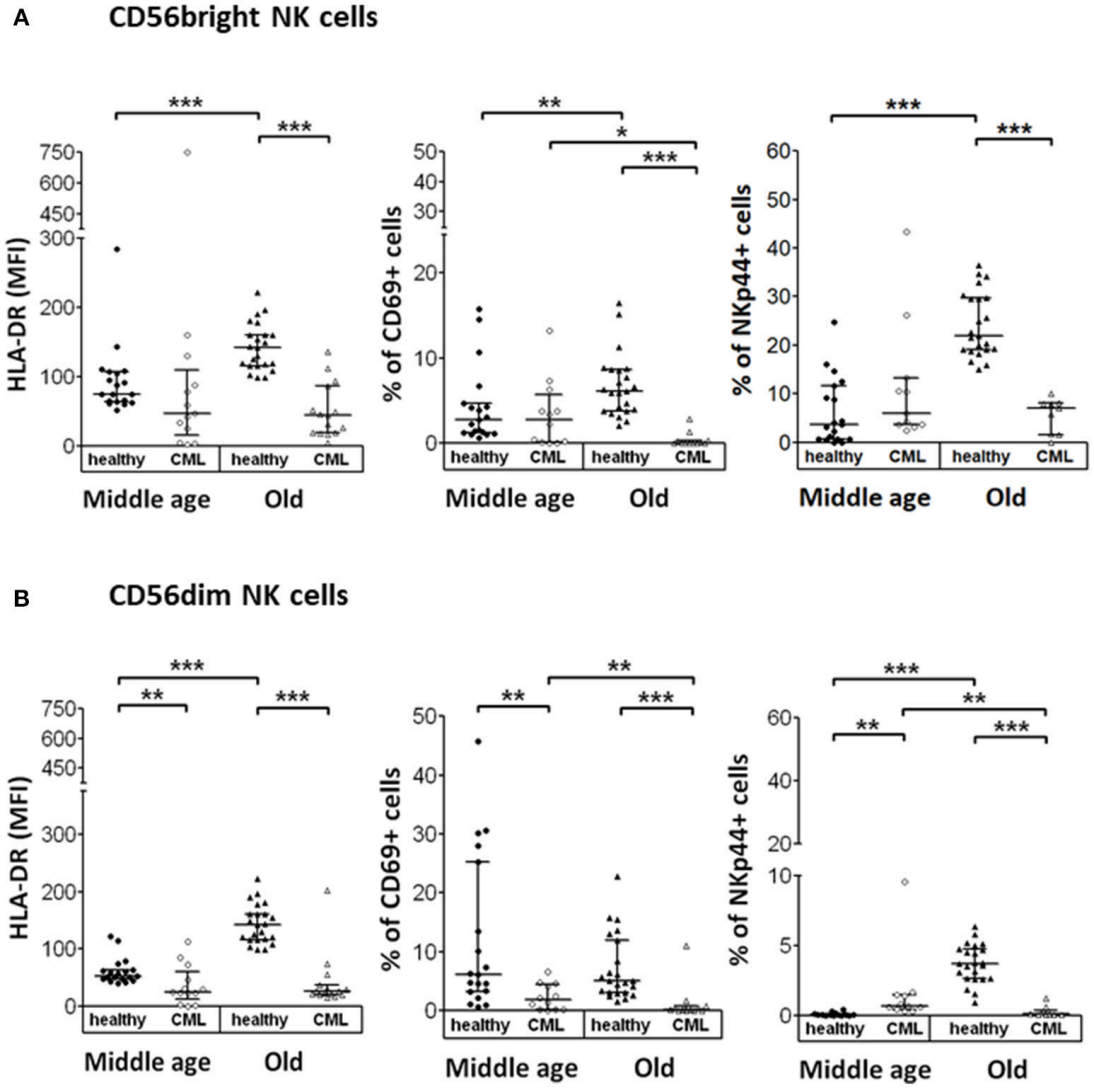
Expression of activation markers on CD56^bright^ and CD56^dim^ NK cell subpopulations. Determination of activation markers expression (HLA-DR, CD69, and NKp44) on the surface of CD56^bright^ and CD56^dim^ NK cells from the different study groups. NK cells were not stimulated. **(A)** Expression of HLA-DR (measured as MFI), CD69 and NKp44 (in percentage) on CD56^bright^ NK cells. **(B)** Expression of HLA-DR (MFI) and percentage of CD69 and NKp44 on CD56^dim^ NK cells. Number of donors: HLA-DR middle-aged healthy *n* = 19, middle-aged CML *n* = 13, old healthy *n* = 23 and old CML *n* = 15; CD69 middle-aged healthy *n* = 19, middle-aged CML *n* = 12, old healthy *n* = 23, and old CML *n* = 15; NKp44 middle-aged healthy *n* = 19, middle-aged CML *n* = 11, old healthy *n* = 23, and old CML *n* = 9. The results, expressed as median with interquartile range, were considered significant at *p* < 0.05. *P-*values were determined comparing middle age with old and healthy with TKI-treated CML patients. ^*^p < 0.05; ^**^p <0.01; ^***^p <0.001.

In this study, we have analyzed the expression of several activation markers (HLA-DR, CD69, and NKp44) on the surface of NK cell subsets in resting conditions. CD56^bright^ NK cells in elderly CML patients showed a decreased expression of HLA-DR (measured as MFI) and a reduction in the percentage of CD69 and NKp44 positive cells compared with elderly healthy donors. An age-associated increase in the expression of these three markers was observed on CD56^bright^ NK cells in elderly healthy donors compared with middle-aged healthy donors. In contrast, in CML patients a decrease of CD69 expression on CD56^bright^ NK cells was associated with age (Figure 3A).

Regarding the expression of these activation markers on the CD56^dim^ NK cell subset, our results showed a decreased expression of these markers in elderly CML patients, as well as a decreased expression of CD69 and HLA-DR in middle-aged CML patients compared with age-matched healthy donors. Nevertheless, it is interesting to highlight the increase observed in the percentage of NKp44^+^ cells in middle-aged CML patients compared with middle-aged healthy donors. We also found an age-associated increase in the expression of HLA-DR and NKp44 in healthy individuals and a decrease of CD69 and NKp44 expression in CD56^dim^ NK cells from elderly CML patients compared with CD56^dim^ NK cells from middle-aged CML patients (Figure 3B).

### Analysis of NK cell differentiation markers on NK cells from TKI-treated CML patients

**Figure 4:**
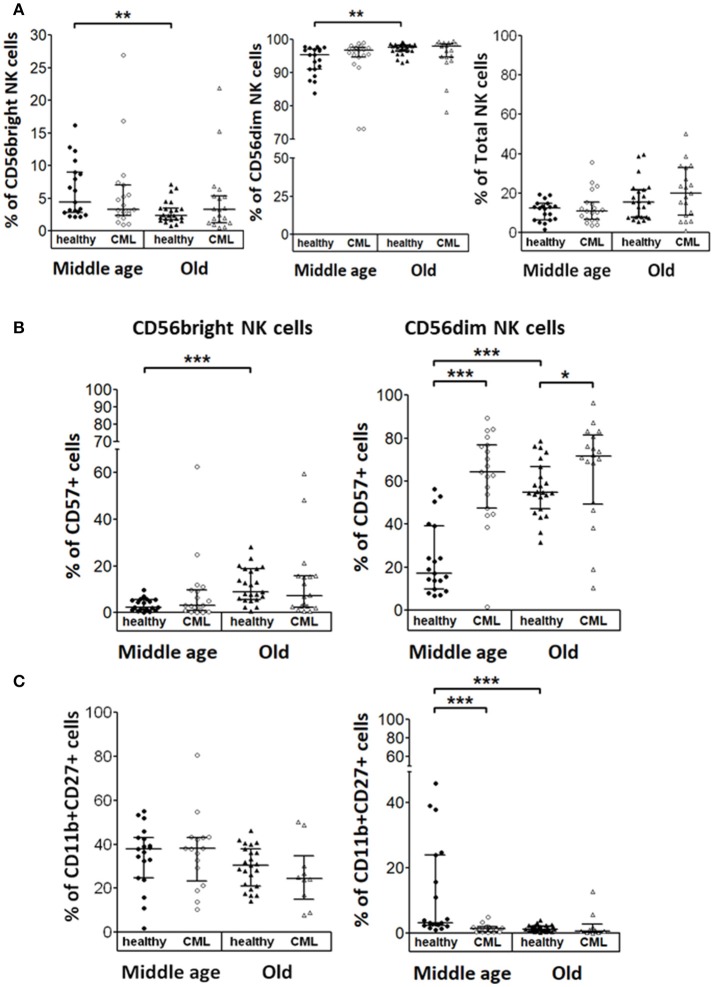
NK cell subsets in healthy and TKI-treated CML individuals stratified by age. **(A)** Percentage of CD56^bright^ and CD56^dim^ NK cells referred to the total of CD56^+^ NK cells and NK cells referred of total Lymphocytes from healthy donors and TKI-treated CML patients. **(B)** Percentage of NK cells expressing CD57 determined in CD56^bright^ and CD56^dim^ NK cell subsets from both healthy and TKI-treated CML individuals. **(C)** Percentage of CD11b and CD27 positive cells in CD56^bright^ and CD56^dim^ NK cell subsets from both healthy and TKI-treated CML individuals. Number of donors: CD56^bright^, CD56^dim^, and NK cells, middle-aged healthy *n* = 19, middle-aged CML *n* = 19, old healthy *n* = 23, and old CML *n* = 19; CD57 middle-aged healthy *n* = 19, middle-aged CML *n* = 19, old healthy *n* = 23, and old CML *n* = 18; CD11b^+^CD27^+^ middle-aged healthy *n* = 19, middle-aged CML *n* = 16, old healthy *n* = 23, and old CML *n* = 10. Graphics show the median with interquartile range. Kruskal-Wallis test was used for multiple comparisons and the Mann-Whitney *U*-test for the comparison of specific groups. The differences were considered significant in a range of *p* < 0.05. ^*^p < 0.05; ^**^p < 0.01; ^***^p < 0.001.

The analysis of NK cell subsets in healthy donors showed a decreased percentage of CD56^bright^ that correlated with an increased percentage of CD56^dim^ NK cells in elderly donors compared with middle-aged healthy donors. In contrast, no statistical significant differences in NK cell subset distribution was observed in CML patients according to age. Percentage of total NK cells was not influenced by CML or age (Figure 4A). The expression of CD57 was increased on CD56^dim^ NK cells from CML patients compared with age-matched healthy donors. Moreover, in healthy individuals, CD57 expression on both CD56^bright^ and CD56^dim^ NK cells increased with age (Figure 4B). The co-expression of CD11b and CD27 markers was also analyzed in each NK cell subset (Figure 4C). Whereas, a high percentage of CD56^bright^ NK cells were CD11b^+^CD27^+^, the majority of CD56^dim^ NK cells from peripheral blood were CD11b^+^CD27^−^. Our results revealed a decrease of CD11b^+^CD27^+^CD56^dim^ NK cells associated with CML in middle-aged individuals and a decrease of this cell subset related to age in healthy donors (Figure 4C). Not significant differences were found in CD56^bright^ NK cells.

### CD107a expression and IFN-γ production in NK cells from TKI-treated CML patients activated with K562

**Figure 5:**
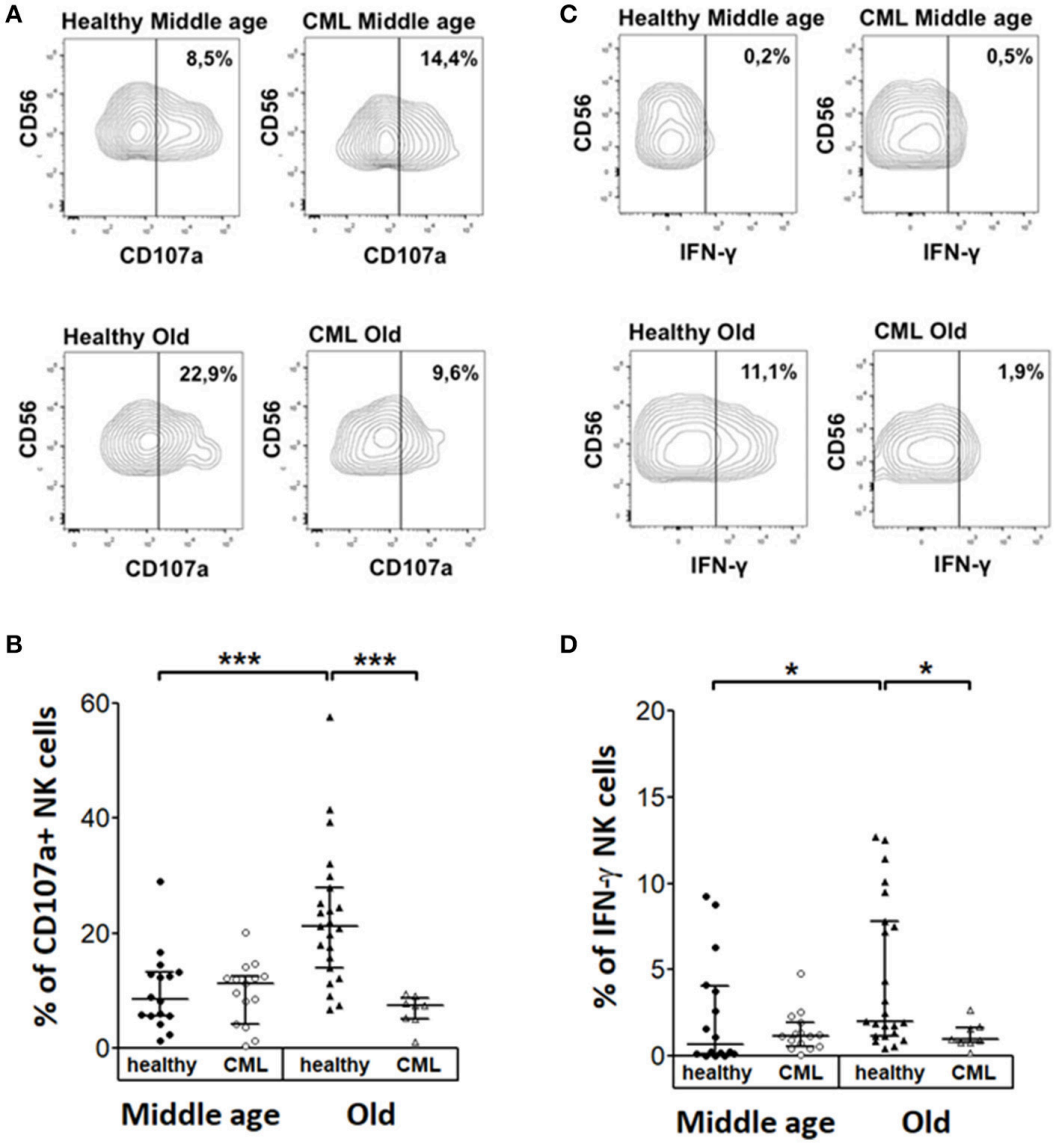
Cytokine expression and degranulation in stimulated NK cells. Analysis of CD107a degranulation assay and IFN-γ expression by Natural killer cells following stimulation with K562 target cells. **(A)** Representative dotplots and **(B)** percentage of CD107a in NK cells from healthy donors and TKI-treated CML patients according to age and CML. **(C)** Representative dotplots and **(D)** IFN-γ expression in NK cells from healthy donors and TKI-treated CML patients according to age and CML (middle-aged healthy *n* = 16, middle-aged CML *n* = 15, old healthy *n* = 23, and old CML *n* = 8). Numbers in **(A,C)** represent the percentage of positive cells referred to the CD56^+^ NK cells. The results in **(B,D)** were shown as median with interquartile range. Values of *p* < 0.05 were considered significant. *P-*values were determined comparing middle age with old and healthy with TKI-treated CML patients. ^*^p < 0.05; ^***^p < 0.001.

We analyzed the percentage of NK cells expressing CD107a or IFN-γ after stimulation with the K562 cell line. Representative analysis of middle-aged and old controls and TKI-treated CML patients are shown Figures 5A,C. The results did not show significant differences on CD107a expression and IFN-γ production in K562 stimulated NK cells between middle-aged healthy donors and middle-aged CML patients. NK cells from healthy elderly donors have higher expression of CD107a or IFN-γ compared with middle-aged healthy donors. In a similar way the percentage of NK cells expressing CD107a or IFN-γ was higher in healthy elderly donors than in TKI-treated CML old patients. On the contrary no significant age-associated differences on CD107a expression and IFN-γ production were observed in K562 stimulated NK cells from CML patients (Figures 5B,D).

As shown in Figure S2, the comparison of cytokine production in healthy controls vs. TKI-treated CML patients shows that the expression of IFN-γ by unstimulated NK cells is higher in middle-aged TKI-treated CML patients and lower in elderly patients compared with their age-matched controls, whereas the expression of IL-10 is higher in TKI-treated CML patients from both age groups compared with their respective controls.

## Discussion

A decrease in the frequency and function of NK cells in CML patients at the time of diagnosis has been demonstrated, with a progressive functional deterioration during disease progression to advanced and blast crisis phase (36–39, 52). In addition, NK cells from CML patients at diagnosis show a reduced expression of activating and inhibitory NK receptors compared to healthy donors (40, 53). NK cells play an important role in the control of CML not only during TKI treatment (40, 41) but also after TKI cessation (54, 55).

**Figure 6:**
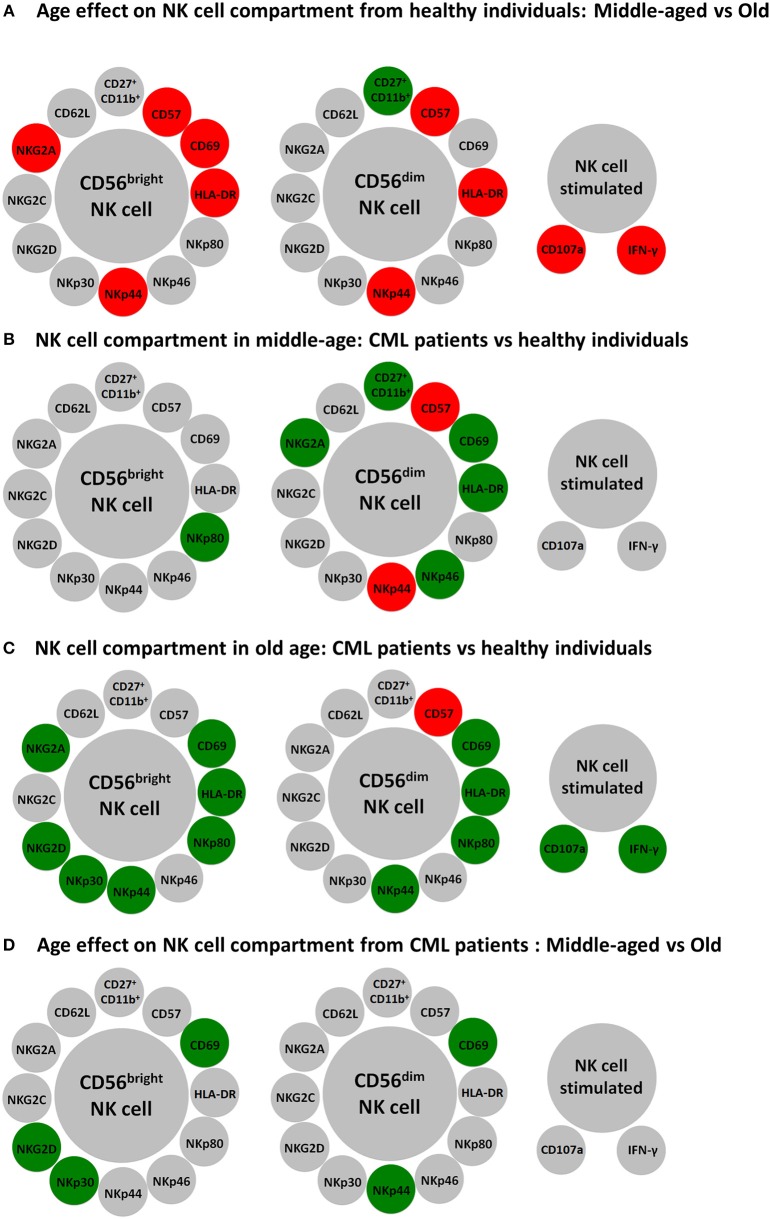
Schematic representation of major differences in resting and K562 stimulated NK cells according to age and CML. Circles represent NK cell subpopulations expressing the indicated membrane markers in CD56^bright^ and CD56^dim^ resting NK cells and the expression of CD107a and IFN-γ in K562 stimulated NK cells. Red color was used for increased expression and green color for decreased expression of the membrane markers in NK cells from healthy old compared with healthy middle-aged individuals **(A)**, from CML patients compared with age-matched healthy donors **(B,C)**, or in old CML patients compared with middle-aged CML patients **(D)**.

It has been shown that life expectancy is lower in older than in younger patients with chronic phase of CML and that aging is a poor prognostic factor for survival and response to treatment in CML (10, 11, 15, 16). Cumulative evidences support that aging affects NK cell subsets, phenotype and function (22, 23, 56), including the expression of activating and inhibitory NK cell receptors (28–30, 57). The analysis of NK cells from middle-aged and elderly healthy donors, summarized in Figure 6A, is in line with previous data on the differences between NK cells from young and old healthy donors. Thus, there are significant differences in the frequency of NK cells subsets with different maturation stages. The percentages of more immature CD56^bright^ NK cells (2, 3), and CD11b^+^/CD27^+^ NK cells, that represent a minor subset of immunoregulatory NK cells described as an intermediate differentiation stage (58), are lower in elderly than in middle-aged healthy donors, whereas the percentage of mature highly cytotoxic CD56^dim^CD57^+^ NK cells is higher in the elderly (Figure 6A), confirming the age-associated shaping of NK cell subsets (22, 30). The expression of NK cell activation markers HLA-DR, CD69, and NKp44 is also higher in NK cells from elderly healthy donors, likely as a consequence of low grade age-associated inflammation (inflamm-aging) (59). Inflamm-aging is also consistent with the observation that NK cells from old healthy donors show higher expression of CD107a or IFN-γ in response to K562 stimulation than NK cells from middle-aged donors. The effect of aging on NK cell cytotoxicity and IFN-γ has been extensively analyzed in discrepant results have been found among different groups probably due to different selection age ranges, technical procedures, and health status of the individuals studied although it is generally accepted that the total number and cytotoxic function of NK cell are preserved or increased in healthy aging compared with young and middle-aged individuals (22, 23, 56).

The study of NK cells in middle-aged TKI-treated CML patients shows that the percentage of CD56^bright^ NK cells is similar to the percentage found in heathy middle-aged individuals, whereas the minor population of CD56^dim^ NK cells co-expressing CD11b and CD27 is dramatically decreased (Figure 6B). In addition, CD56^dim^ NK cells from TKI-treated CML patients have higher expression of CD57, a marker of highly differentiated NK cells (22, 30), and NKp44 indicating that NK cells from TKI-treated CML patients are highly differentiated activated NK cells, as it has been recently suggested (40, 54). However, there is a lower expression of NKp46, NKG2A, CD69, and HLA-DR on CD56^dim^ NK cells and of NKp80 on CD56^bright^ NK cells (Figure 6B). The comparison of NK cells from old TKI-treated CML patients with those from old healthy individuals shows a lower expression of NK receptors NKp44, NKp80, CD69, and HLA-DR in both CD56^bright^ and CD56^dim^ NK cells and also a reduced expression of NKp30, NKG2D, and NKG2A in CD56^bright^ NK cells (Figure 6C). Thus, despite the well-established observations that CML patients have a decreased expression of NK receptors at diagnosis (36–39), our results confirm that NK cells from TKI-treatment CML patients can express NK activating receptors (40, 41) such as NKp30, NKp46, and NKp80, although this expression is heterogeneous and in some cases their levels are lower than those found in age-matched healthy controls. NKp30 and NKp46 are NCRs involved in NK cells cytotoxicity after interaction with their ligands on target cells (60). NKp80, is an activating C-type lectin-like receptor expressed on NK cells that interact with its ligand activation-induced C-type lectin (AICL) expressed on myeloid cells, including myeloid leukemia cells (61). It has been suggested that after TKI treatment NK cells are involved in the control of CML blasts (40, 41), thus the lower expression of these receptors compared with healthy controls can be the consequence of the interaction of NK cells with their ligands expressed on leukemic blasts, as suggested for NK cells from AML patients (44–46). Downregulation of NKG2D after its interactions with MICA/B ligand is a well-defined phenomenon in different tumors (62, 63). However, the expression of NKG2D is well preserved in all NK cell subsets from TKI treated CML patients, with the exception of CD56^bright^ from elderly patients, confirming recent studies showing that the downregulated expression of NKG2D at the time of CML diagnosis, is restored to normal levels after TKI treatment (40, 41). The comparison of NK cell response to K562 in healthy controls vs. TKI-treated CML patients did not show significant differences on CD107a expression in K562 stimulated NK cells when middle-aged healthy donors were compared with middle-aged or old CML patients (Figures 6B,C) supporting previous findings that TKI treatment is associated with immune system re-activation and restoration of NK cell immune surveillance in CML patients (40, 41). On the contrary the observation that the percentage of NK cells expressing CD107a in TKI-treated CML old patients was lower than in healthy elderly donors, together with the lower expression of NK receptors in old TKI-treated CML patients compared with healthy elderly healthy donors (Figure 6C), support that the alterations on NK cells observed in healthy elderly donors likely associated with chronic virus infection, such as CMV, and inflamm-aging are not observed in old TKI-treated CML patients. The high variability observed in IFN-γ production by NK cells in response to K562 in healthy donors and the low response observed in most CML patients, represent a limitation of the study that precludes to obtain a conclusion on the significance of cytokine production in the disease control. The NK cell functional capacity and cytokine production during TKI-treatment and after TKI cessation in CML patients requires further analysis to discriminate a possible role in the long-term elimination of CML blasts (54, 55).

The analysis of the possible effect of age on NK cells from TKI-treated CML patients shows a lower expression of activation markers NKp44 and CD69 in elderly compared with middle-aged TKI-treated CML patients whereas no significant differences related with age are found in the other parameters studied, including CD107a expression and IFN-γ production in K562 stimulated NK cells from TKI-treated CML patients (Figure 6D), indicating that age is not a limitation of the NK cell recovery after treatment with TKI.

In conclusion, despite the deleterious effect of aging in CML prognosis, our results showing that activating NK cell receptors can be expressed both in middle-aged and elderly TKI-treated CML patients highlight the interest to extensively analyse the effect of aging on NK cell phenotype and function in these patients. The possibility of enhancing NK cell activity by using cytokines and immunomodulating agents open new perspectives for the design of novel clinical trials aiming effective long-term treatment-free remission after TKI cessation in CML patients.

## Author contributions

PR-S, NL-S, CC, RS, RT, and MS-R: research study design; PR-S, NL-S, JSA, PC, and VA: experiments conduction and data acquisition; LR and PF-T: clinical data and patient management; PR-S, NL-S, RT, JSA, CC, and CA: data analysis; PR-S, CA, and MS-R: reagents providing; PR-S, NL-S, CC, RT, and RS: manuscript writing; All authors approved the final version of the manuscript.

### Conflict of interest statement

The authors declare that the research was conducted in the absence of any commercial or financial relationships that could be construed as a potential conflict of interest.
