# Supplementary material for: Effect of Age on NK Cell Compartment in Chronic Myeloid Leukemia Patients Treated With Tyrosine Kinase Inhibitors
Source: Front Immunol. 2018 Nov 8;9:2587. doi: 10.3389/fimmu.2018.02587 (PMC6246921; doi:10.3389/fimmu.2018.02587)
Supplement: Supplementary file 1 [file Image_1.pdf]

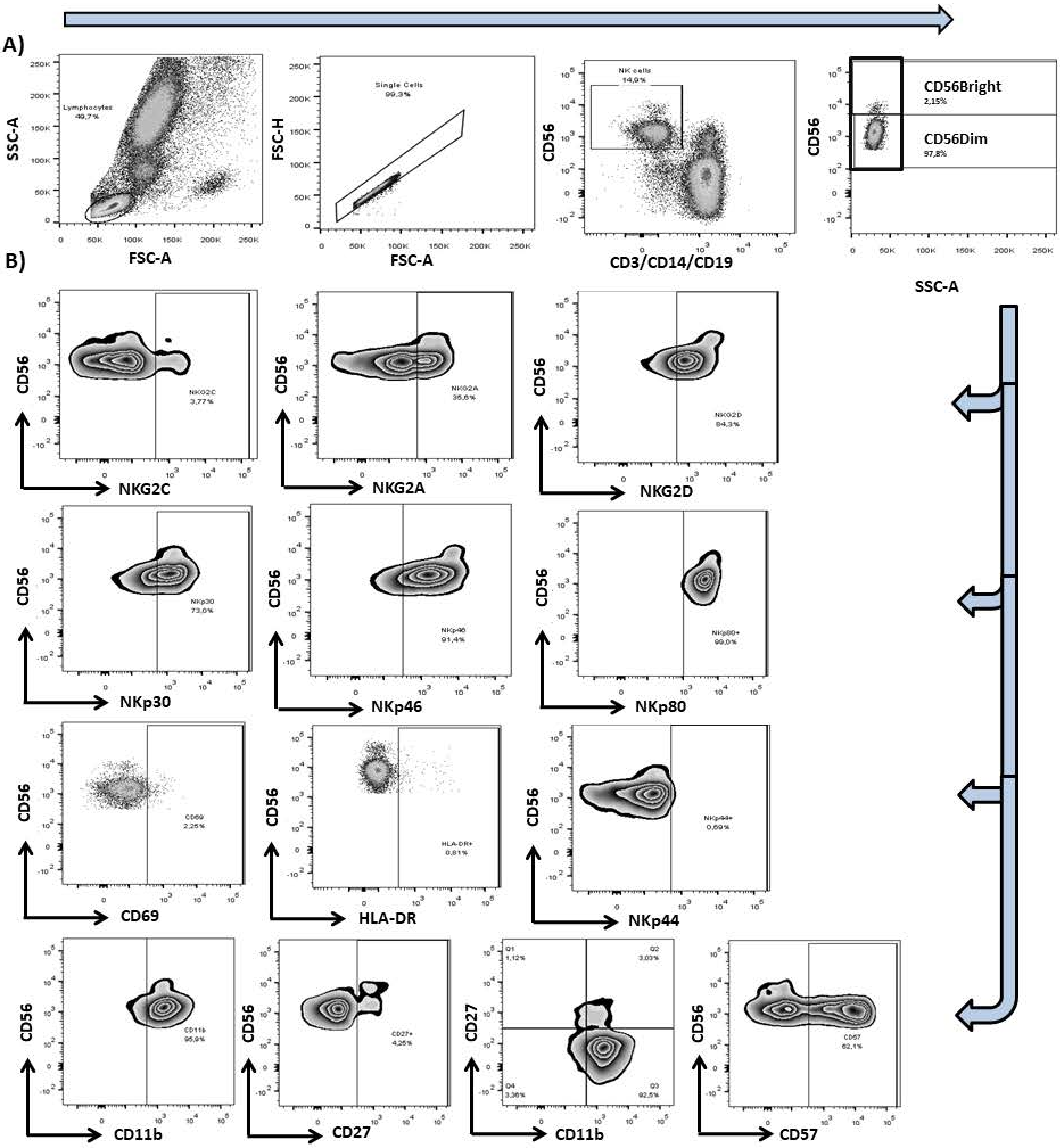

Figure S1A. Gating strategy used for the analysis NK cell markers. Lymphocytes were selected from total cells, doublets were removed using FSC-A and FSC-H, the dump channel (CD3/CD14/CD19) was excluded and finally NK cells were selected according to the CD56 expression. **B)** Receptors studied in the subpopulations CD56bright and CD56dim NK cells.

B)

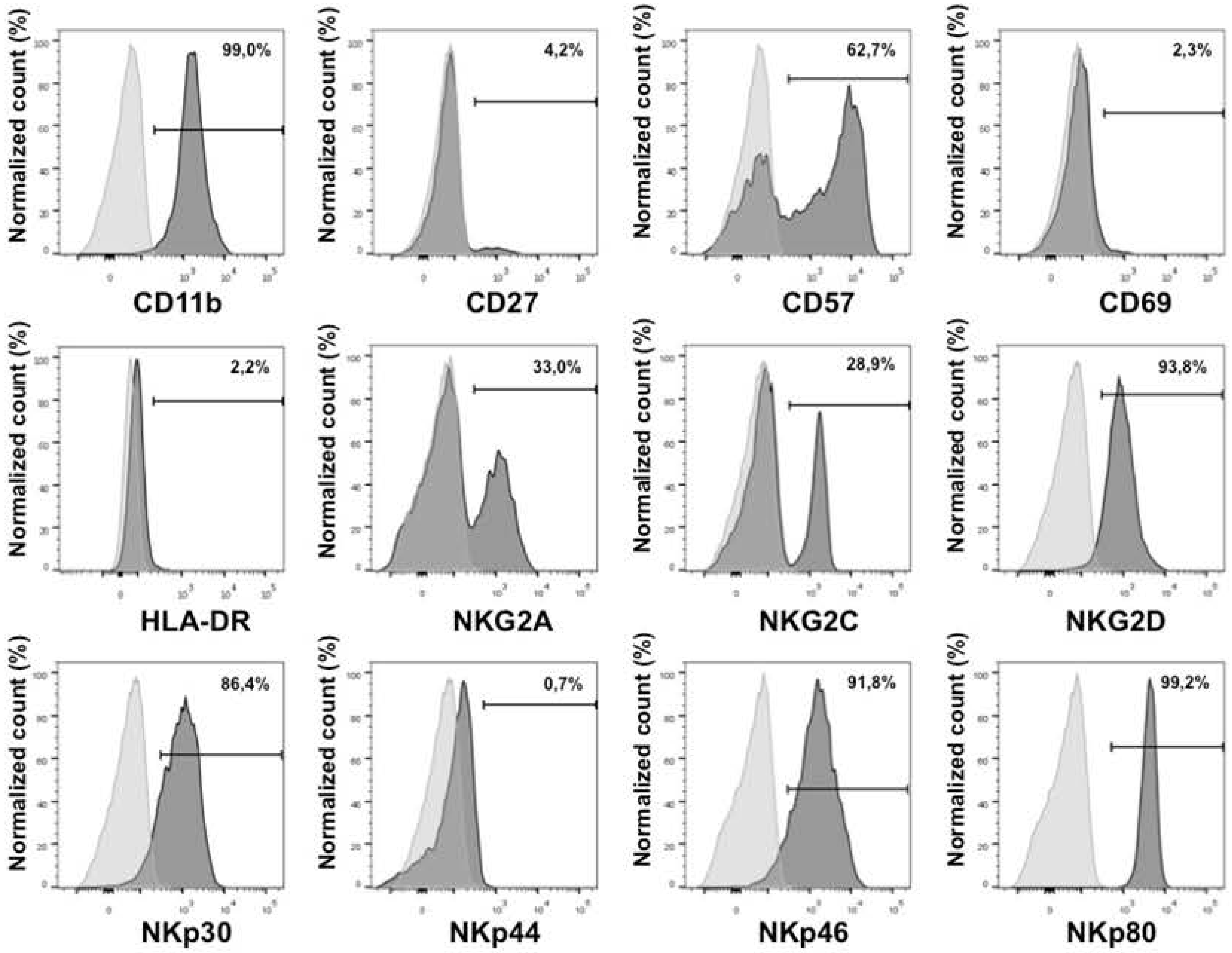

Specificity	Fluorochrome	Isotype
CD11b	V450	IgG1 κ
CD27	FITC	IgG1 κ
CD57	PacificBlue	IgM κ
CD69	FITC	IgG1 κ
HLA-DR	V500	IgG2a κ
NKG2A	PE	IgG2a κ
NKG2C	APC	IgG1 κ
NKG2D	APC	IgG1 κ
NKp30	AF647	IgG1 κ
NKp44	AF647	IgG1 κ
NKp46	PE	IgG1 κ
NKp80	PE	IgG1 κ

Figure S1B. Representative histograms used for analysis of NK cell markers. Representative histograms for each marker are shown in the upper part of the figure. The light gray area represents the isotype controls and the dark gray area represents the positive region. The antibodies used for the analysis of NK cell markers, indicating the fluorochrome and the isotype, are listed in the lower part of the figure. Isotype matched antibodies labelled with the appropriate fluorochrome were used as negative control.
