# Supplementary figures and images for: Effect of Age on NK Cell Compartment in Chronic Myeloid Leukemia Patients Treated With Tyrosine Kinase Inhibitors
Source: Front Immunol. 2018 Nov 8;9:2587. doi: 10.3389/fimmu.2018.02587 (PMC6246921; doi:10.3389/fimmu.2018.02587)

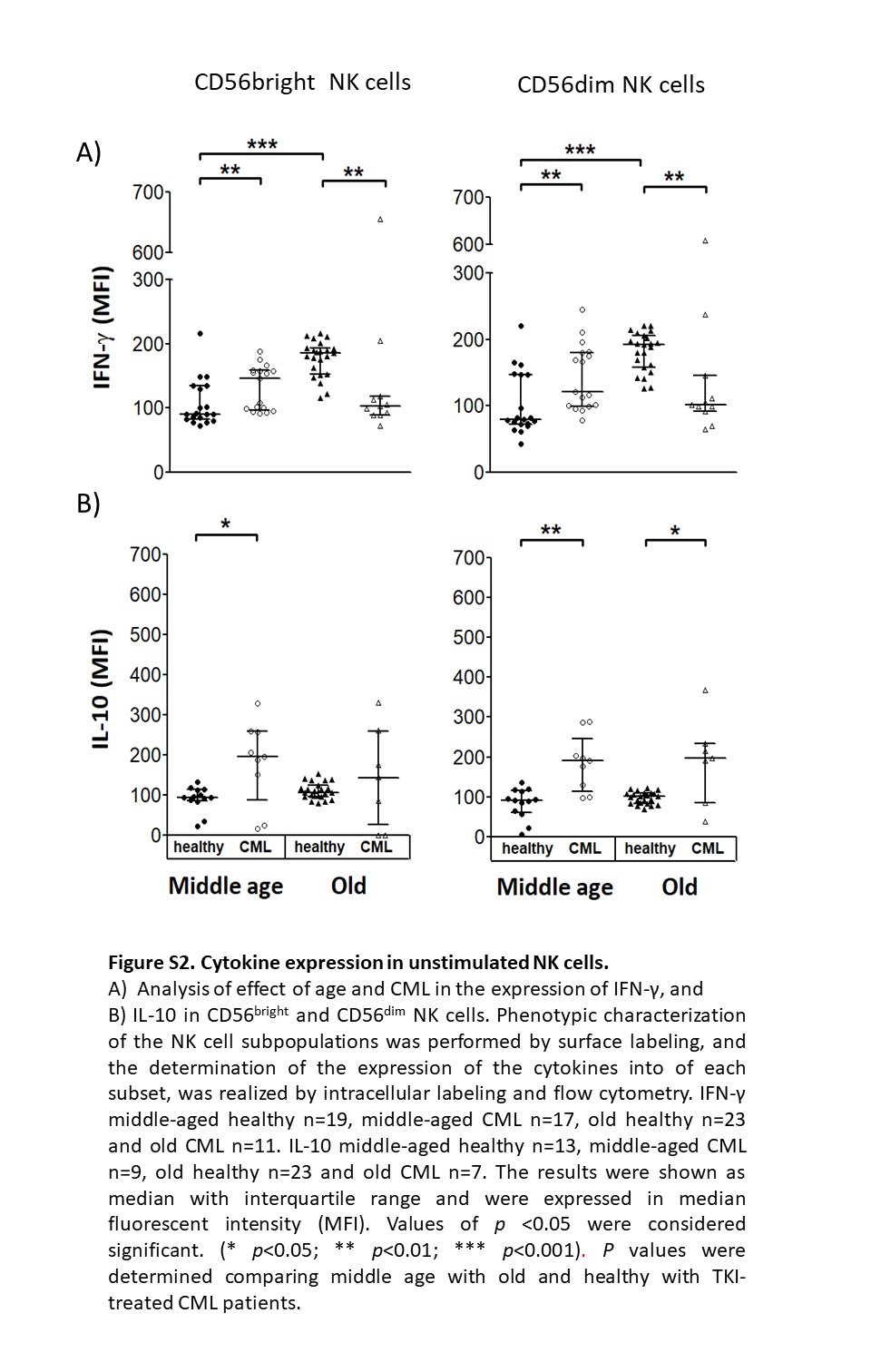

Supplement: Supplementary file 2 [file Image_2.tif]
